# Peer support to maintain psychological wellbeing in people with advanced cancer: findings from a feasibility study for a randomised controlled trial

**DOI:** 10.1186/s12904-020-00631-z

**Published:** 2020-08-17

**Authors:** Catherine Walshe, Diane Roberts, Lynn Calman, Lynda Appleton, Robert Croft, Suzanne Skevington, Mari Lloyd-Williams, Gunn Grande, Guillermo Perez Algorta

**Affiliations:** 1 International Observatory on End of Life Care, Division of Health Research, Lancaster University, Bailrigg, Lancaster, UK; 2 Division of Nursing, Midwifery and Social Work, Manchester University, Manchester, UK; 3 Macmillan Survivorship Research Group, School of Health Sciences, Southampton University, Southampton, UK; 4 Clatterbridge Cancer Centre NHS Foundation Trust, Wirral, UK; 5 Liverpool, UK; 6 Manchester Centre for Health Psychology, School of Psychological Sciences, Manchester University, Manchester, UK; 7 APSCSG, Institute of Population and Health Sciences, Liverpool, UK; 8 Division of Health Research, Lancaster University, Lancaster, UK

**Keywords:** Cancer, Peer support, Feasibility study, Palliative care

## Abstract

**Background:**

Advanced cancer affects people’s lives, often causing stress, anxiety and depression. Peer mentor interventions are used to address psychosocial concerns, but their outcomes and effect are not known. Our objective was to determine the feasibility of delivering and investigating a novel peer mentor intervention to promote and maintain psychological wellbeing in people with advanced cancer.

**Methods:**

A mixed methods design incorporating a two-armed controlled trial (random allocation ratio 1:1) of a proactive peer mentor intervention plus usual care, vs. usual care alone, and a qualitative process evaluation. Peer mentors were recruited, trained, and matched with people with advanced cancer. Quantitative data assessed quality of life, coping styles, depression, social support and use of healthcare and other supports. Qualitative interviews probed experiences of the study and intervention.

**Results:**

Peer mentor training and numbers (*n* = 12) met feasibility targets. Patient participants (n = 12, from 181 eligible who received an information pack) were not recruited to feasibility targets. Those who entered the study demonstrated that intervention delivery and data collection were feasible. Outcome data must be treated with extreme caution due to small numbers, but indicate that the intervention may have a positive effect on quality of life.

**Conclusions:**

Peer mentor interventions are worthy of further study and researchers can learn from these feasibility data in planning participant recruitment and data collection strategies. Pragmatic trials, where the effectiveness of an intervention is tested in real-world routine practice, may be most appropriate. Peer mentor interventions may have merit in enabling survivors with advanced cancer cope with their disease.

**Trial Registration:**

The trial was prospectively registered 13.6.2016: ISRCTN10276684.

## Background

The diagnosis of advanced cancer and its effects can impact negatively on the everyday lives of people with cancer and those who support them. Depression and anxiety are common in those with advanced cancer [1–3], and depression severity is a strong predictor of poor quality of life [4]. Pharmacological interventions are common, but with little evidence of effect [5]. Some non-pharmacological interventions show promise in enabling people with advanced cancer to maintain or regain psychological wellbeing [6, 7]. A commonly used, but under-researched intervention is the use of peer support [8].

Peer support involves people drawing on shared personal experience to provide knowledge, social interaction, emotional assistance or practical help, often in a way that is mutually beneficial. The theoretical mechanisms proposed to underpin effective peer support include experiential knowledge, social support, social comparison and the helper therapy principle [9]. Offering support to those in similar situations appears to be an inbuilt human response, seen more broadly in initiatives such as compassionate communities [10], and where such human interaction is in itself likely to be of benefit to both those volunteering support and those receiving it [11–13].

Peer support is often called a ‘created’ social network, provided with a range of professional support and involvement, ranging from self-help groups with little outside involvement to ‘paraprofessionals’ who may have extensive training for their peer support role [14]. Whilst peer mentoring is often offered in group settings, there is a developing focus on internet facilitation [15, 16], as well as one-to-one support [17].

Studies describe benefits and popularity of peer support to people with cancer [18, 19]. However there are few robust trials, and no data that allow a determination of the best form of peer support for people with advanced cancer [8]. Such interventions may have promise, as they have effects where used in those with other diseases, and an effect demonstrated from less targeted befriending interventions [20, 21].

We previously conducted a qualitative study that demonstrated that people with advanced cancer can, and do, cope well at times, using a range of strategies to enable this coping, and to maintain their own wellbeing. They expressed a preference for one-to-one, face-to-face peer mentoring as a form of peer support, to enable them to learn from these coping strategies [22, 23]. This study tests the feasibility of conducting a randomised controlled trial of a novel peer mentor intervention in which the content (coping strategies to maintain wellbeing), and chosen delivery mechanism (via trained peer mentors) are both derived from our previous qualitative study [22, 23].

## Methods

### Objectives

The aim of this study is to determine the feasibility of delivering and investigating a novel peer mentor intervention to promote and maintain psychological wellbeing in people with advanced cancer. The focus is on the feasibility of delivering the intervention in a trial context, and determining appropriate study design choices and parameters to maximise the probability of a well-run and adequately powered future trial. Specific objectives focus on intervention delivery and investigation:

INTERVENTION DELIVERY OBJECTIVES

i. Developing a clear peer mentor intervention specification, acceptable to both peer mentors and patients with advanced cancer in the context of a randomised controlled trial.
ii. Understanding the operational implications of running, monitoring and maintaining a proactively delivered peer mentor scheme to convey novel evidence-based information as part of a randomised controlled trial.
iii. Understanding how peer mentors deliver the intervention and information about maintaining wellbeing to people with advanced cancer.

INTERVENTION INVESTIGATION OBJECTIVES

iv. Calculating the number of patient and mentor participants needed to power a full trial of the intervention.
v. Understanding issues of recruitment, retention and attrition of peer mentors and patient participants in the context of planning a full trial.
vi. Exploring the impact of randomisation within a volunteer-delivered intervention.
vii. Understanding potential intervention contamination between the trial arms, or from alternative sources of information on coping strategies.
viii. Determining the type and consequences of any adverse events or serious adverse events.
ix. Confirming primary and secondary outcome measures, developing a time schedule for their administration (recognising attrition), determining the time point for primary outcome (4 weeks or 12 weeks)
x. Determining our ability to collect data on complex health service use (service referral, GP visits, use of psychological support services, prescription of anti-depressants) for a health economics component in any future full trial.

### Design

This study employed a mixed methods design incorporating a two-armed controlled feasibility trial with a 1:1 random allocation ratio, of a proactive peer mentor intervention plus usual care vs. usual care alone, with a qualitative process evaluation. The study is reported following CONSORT guidelines for pilot and feasibility studies [24]. The study was prospectively registered: ISRCTN10276684.

### Participants

Three types of participants were recruited for the study: people with advanced cancer (patients), their family/informal carers, and peer mentors. For patients and carers, these criteria were the same as those used in our earlier qualitative study [22, 23]. The inclusion and exclusion criteria were:

People with advanced cancer:

- Adults > 16 years. No upper age limit.
- With advanced cancer (any type), defined as metastatic disease at diagnosis, and/or with local or metastatic spread following treatment, and/or where prognosis is estimated as less than a year.
- Those whom their health care professionals judge to have a prognosis > 3 months, to facilitate study completion.
- Those whom their health care professionals judge have capacity to give informed consent to research participation.
- Assessed by their health care professional as understanding their diagnosis of advanced cancer.
- Able to adequately understand and respond to verbal and written material in English.

Informal/family carers of recruited patients:

- Adults > 16 years. No upper age limit.
- Provider of informal support to a person with advanced cancer.
- Able to adequately understand and respond to verbal and written material in English.

Excluded: Paid or professional carers for the person with advanced cancer.

Peer mentors:

- Experience of living with cancer, but at least 6+ months post diagnosis.
- Age 18+
- Able to commit to 6 months of volunteering.
- Have 2h hours per week available for volunteering.
- Live in the geographic area selected for the project.
- e fluent in written and spoken English.
- Able to demonstrate empathetic communication skills during peer mentor training.
- Satisfactory completion of project-specific peer mentor training.
- Disclosure and Barring Service (DBS) clearance for working with vulnerable people.

### Settings

Patient participants were principally recruited via cancer centres in North West England, although recruitment via hospices was possible towards the end of the study. The setting for the peer mentor intervention was in community (e.g. café) or home settings.

### Recruitment

People with advanced cancer were recruited through oncology or palliative care clinics situated in NHS cancer centres and/or local hospices. Clinical staff and/or research nurses were responsible for identifying those who met the inclusion criteria, and then offering them a participant recruitment pack (easy-read leaflet, information sheet, reply slip). Potential participants then responded to the research team (telephone, post, email) to indicate if they were interested in participating, or not. Approximately half way through the study a protocol amendment enabled follow up phone calls to those who had taken a recruitment pack but who had not yet responded to the research team within 2 weeks. Checks were in place between the research and clinical teams to ensure no contacts were made with potential participants who may be too unwell to participate. Patient participants were asked to pass on a carer recruitment pack to ‘the person who provides them with most support’. Patient participants were not required to recruit a carer participant as a prerequisite for study participation. Once potential participants had given assent to the research team contacting them, a home visit was arranged, and written informed consent obtained by a member of the research team.

Peer mentor participants were recruited via advertising within the cancer centres, through local media, volunteer bureaux, websites (http://www.do-it.org.uk/) and social media. They contacted the research team, received information about the study, and were invited to peer mentor training. Final written informed consent was taken on completion of the training and receipt of Disclosure and Barring Service checks. Further information on their recruitment, training and flow through the study is provided in a partner publication [25].

### Sample size

A sample size of 33 patients per arm of the trial was estimated to be required based on feasibility study literature [26], and attrition from our past qualitative study of 0% at 4 weeks and 7.5% at 12 weeks [22, 23]. We anticipated training 12–15 peer mentors.

### Intervention description

The intervention consists of the proactive introduction to, and informal contact with a trained peer mentor for people with advanced cancer. Potential peer mentors were assessed and disclosure and barring service (DBS) checking and attended a 2-day training session. Training covered standard items such as safety, risk assessment, and lone working, as well as bespoke sessions on ways of coping with cancer shown from our prior research [22]. As patient participants were recruited, they were matched to peer mentors by the research team based on any explicit requests (e.g. gender) and locality. The intervention lasted ≤12 weeks, with earlier termination in the case of death, illness precluding participation, or request. Trained peer mentors initiated informal contact with the patient ≤2 times per week, either face-to-face or by telephone. The content of each contact was individually tailored to individual needs and circumstances, but was capped at ≤2 h. Face-to-face interactions were in informal settings such as patients’ homes or shared public informal spaces (e.g. cafes) as chosen by participants. The intervention was delivered alongside ‘usual care’, defined as any health or social care accessed by patient participants during the study. The control group received usual care only. Regular support for peer mentors was available, and an out- of-hours telephone service was available to all participants (mentors and patients). Peer mentors followed agreed protocols for lone working and issue escalation, such as concerns about wellbeing, and were reimbursed for appropriate out-of-pocket expenses incurred in their role as mentor.

### Data collection

Data collection activities were designed to map on to the feasibility objectives, and each is identified with a numeral relating to its feasibility objective (i–x). Quantitative outcome data were collected on three occasions: T1 baseline, T2 4 weeks and T3 12 weeks (or termination of the intervention). Baseline data were collected at a face-to-face meeting. Subsequent quantitative data were collected via postal questionnaires at 4 and 12 weeks.

Mentors kept logs of contacts (visit, call, text), and researchers kept logs of all study activities (recruitment enquiries and activities, peer-mentor training attendance, attrition, data collection completions, progress through the study). Qualitative interviews were requested with mentors, patient and carer participants after the intervention was completed (≤ 12 weeks, or earlier if terminated). These were conducted face-to-face at a place of the participants choosing, and digitally audio-recorded. Processes were in place to prevent contacting people inappropriately e.g. due to death or severe illness.

Data were collected regarding peer mentors via researcher completed study logs and qualitative interviews with peer mentors (recruitment processes, training, attrition, interaction with patients, wellbeing and perceptions of intervention) (i, ii, iii, v, vi, vii viii); about parameters to design a full study via researcher completed study logs and qualitative interviews with peer mentors and patient participants (participant recruitment, trial processes including outcome measure completion, any intervention contamination, and health service usage) (v, vi, vii, viii, x); data from outcome measures for future primary and secondary trial outcomes to facilitate a power calculation/effect size for any future full trial (iv, ix, x).

Our primary outcome for a full trial was anticipated to be patient quality of life, primarily in the psychological domain, at T2 = 4 weeks, assessed by the World Health Organisation Quality of Life short- form assessment (WHOQOL-BREF) (UK version). This timescale allows assessment of the subjective outcomes of the intervention over a limited period of time, which is necessary as life expectancy may be short for some participants. The WHOQOL-BREF is a generic, broad-ranging, validated, quality of life measure that assesses quality of life in four domains; physical, social, psychological and environmental [27, 28]; its scores respond to changes over time [29]. We also collected data anticipated to be secondary outcomes for a full trial, to assess their usage and completion:

- Patient and carer generic quality of life (WHOQOL-BREF) at T3 = 12 weeks (or completion of intervention if before 12 weeks) [27, 28].
- Patient health-related quality of life specifically for cancer (EORTC QLQ C 15 PAL) at T2 = 4 and T3 = 12 weeks (or completion of intervention if before 12 weeks). This is a shorter (15 item) tool validated for palliative care populations, which asks about quality of life issues over the previous week [30, 31]. This allows assessment of the most recent impact of the intervention (over 1 week). The World Health Organisation recommend that both generic and disease specific measures are used.
- Patient and carer coping strategies (using Brief COPE at T2 = 4 and T3 = 12 or sooner). To assess whether the intervention affects coping strategies [32].
- Patient depression using PHQ-9 (a validated short tool, used in people with advanced cancer) at T2 = 4 weeks and T3 = 12 weeks (or sooner) [33].
- Social Support (mMOS-SS [34] patient participants, CSNAT [35] carer participants) measured at T2 = 4 and T3 = 12 (or sooner) to assess changes over the intervention period to understand competing impact of intervention on social support.

Patient, carer and peer mentor participants were at liberty to withdraw without giving a reason, at any time during the study. Any withdrawals such as for distress, or crossing personal boundaries, were monitored.

### Randomisation

Manchester Academic Health Sciences Centre – Clinical Trials Unit (CTU) generated the random allocation sequence, and allocations were made at time of patient participant recruitment with the research team telephoning the CTU to enable disclosure of the allocation after consent and baseline data collection. Carers were not randomised as their initial and continued participation was dependent on patient participation and continuation in the study. Blinding was not possible due to the nature of the intervention, but those entering data were blinded to the allocation.

### Data analysis and statistical methods

The data analysis plan addressed quantitative feasibility data (e.g. attrition rate), the qualitative process evaluation, and analysis of data collected using validated tools to measure proposed future primary and secondary trial outcomes. The data were summarised using means, medians, standard deviations and interquartile range. We also reported the sample score range and missing values. The analysis of the primary endpoint was planned using an independent group t-test on the difference from baseline (T1) to T2 and T3, or the non-parametric equivalent (Mann-Whitney test), but the small sample size meant this was not appropriate. Thematic analysis was used to analyse the data from qualitative, semi-structured interviews. These were fully transcribed, and then thematic analysis followed a staged process of familiarisation, initial code generation, collating codes into potential themes, theme review and naming [36]. NVivo™ software was used to manage data.

### Research ethics and governance approvals

The study was sponsored by Lancaster University, and received NHS Research Ethics Committee approval from Wales REC 5 (16/WA/0032) on 3rd February 2016. All relevant Health Research Authority and governance approvals were gained.

## Results

### Participants

**Fig. 1:**
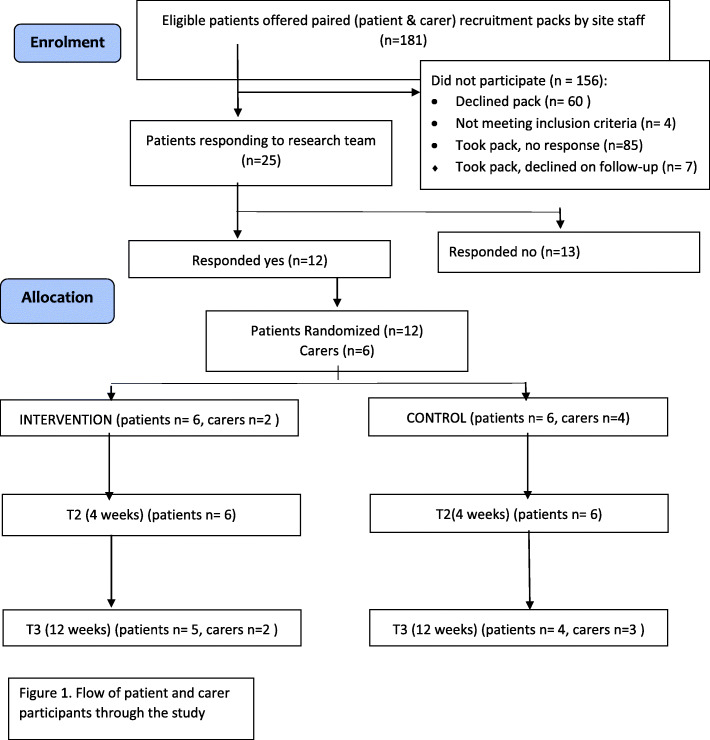
Flow of patient and carer participants through the study

Recruitment of peer mentors to the study commenced in October 2016, with the first training session held in January 2017. Recruitment of patient and carer participants to the study commenced in March 2017, and ceased in April 2018. Patient recruitment to the study is described in Fig. 1, and participant characteristics in Table 1. Qualitative interviews were conducted with four patients, two carers and seven mentors.

**Table 1:** Participant baseline characteristics

General	Patient recruited (Control) (*n* = 6)	Patients recruited (Intervention) (*n* = 6)	Carer recruited (*n* = 6)	Mentor recruited (*n* = 12)
Gender, *Male*, n	4	3	2	4
Age, mean (SD)	69.6 (9.7)	64.8 (6.2)	69.6 (5.6)	60.8 (8.6)
*Diagnosis*:			NA	
Cancer (other)	2	2		6
Bowel cancer	1	1		1
Ovarian cancer	1	–		1
Head and Neck cancer	1	–		–
Prostate cancer	1	2		1
Breast cancer	–	1		3
Ethnicity, *British*, n	6	6	6	11
Marital status, *Married*, n	6	3	6	7
Living status, *Living with partner no children*, n	6	3	6	6
Living status, *Living alone*, n	0	3	0	3
Employment status, *Retired*, n	5	3	6	8
Spirituality, *Religious but not actively engaged in practice*, n	3	3	2	9
Site, *Cancer centre A*^*a*^, n	4	4	4	8

^a^ Successful recruitment was from two cancer centres (A and B)

### Recruitment issues

**Fig. 2:**
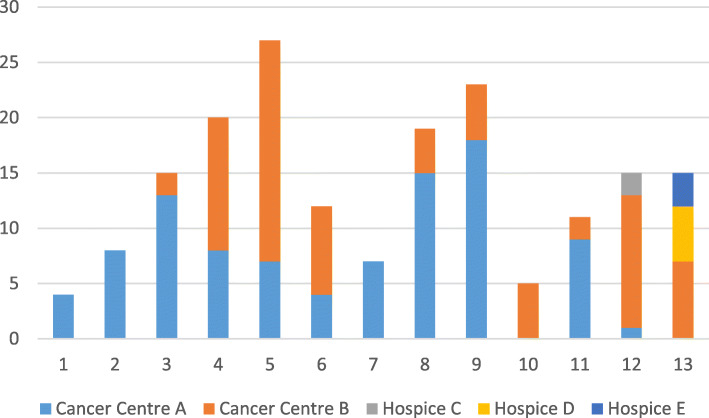
Eligible patients identified, per month, for each recruitment site

**Fig. 3:**
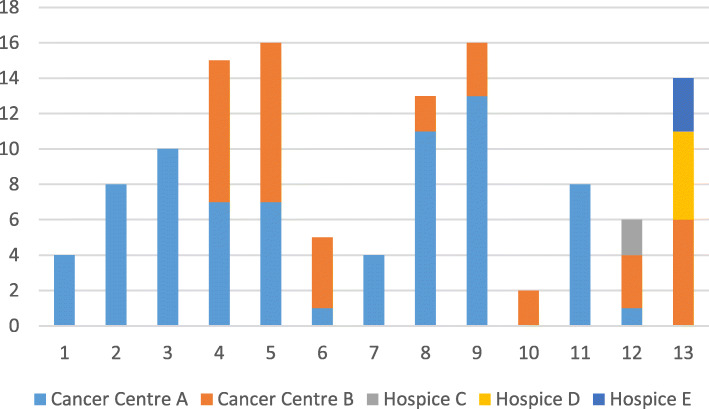
Recruitment packs distributed, per month, for each recruitment site

Recruitment of peer mentors to the study was unproblematic, with good numbers (*n* = 48) responding. Twelve completed training and were available as peer mentors, on target. However, recruitment of patient participants was problematic, with the numbers required to fully assess feasibility not recruited to the study. This then affected the number of paired informal carers recruited. Initial recruitment methods mirrored the effective recruitment plan we had used in our pre-cursor qualitative study, with the same patient inclusion and exclusion criteria [22]. Two issues were apparent. First, the number of packs distributed per month of recruitment to eligible patients were lower than anticipated. Second, the enquiry rate of potential participants who had received a pack was low; this issue was primarily responsible for the low numbers recruited to the study. These data are displayed in Figs. 2 and 3.

There were concerns that recruitment may be challenging because potential participants might find it hard to admit that they needed support, as a mentor reflected on their own experience:

*I didn’t tell the family how bad I felt, but there again I didn’t tell anyone, I kept it to myself. Had I had somebody to go to, to off-load, that might have eased it a little bit. M504*

Active monitoring meant that recruitment challenges were identified early in the study, and a number of changes were made to recruitment processes to attempt to address these. In particular several protocol amendments were made, summarised in Table 2, although these did not prove effective. We extended the recruitment period within the overall study timescale, but could not extend outside the funded study time.

**Table 2:** Study protocol amendments made to address recruitment issues

Concern	Amendment made	Timescale
Clinicians and patients potentially concerned or distressed about the term ‘advanced cancer’ used in the approved (by our PPI and the REC) study recruitment materials.	The term ‘advanced cancer’ removed from materials and replaced with ‘cancer’.	Amendment approved month 5 of recruitment
The study was hard to quickly introduce and explain in a busy clinic environment	A quick-read bi-fold summary leaflet was prepared, worded and laid out according to PPI feedback, attached to the outside of the recruitment pack.	Amendment approved month 5 of recruitment
Most potential participants were taking a recruitment pack in clinic, but not responding to the research team.	Telephone follow up by the research nurse teams at the recruiting sites added.	Amendment approved month 9 of recruitment.
Cancer centre clinics may not be the most appropriate place to recruit participants for this type of study.	Hospices added as recruitment sites	Amendment approved month 11 of recruitment.

### Randomisation

All those who expressed an interest in participation consented to take part, and agreed to the randomisation procedure. There were no adverse comments on the randomisation procedure from participating patients who were subsequently interviewed. However, some of those allocated to the control arm expressed disappointment at not receiving the intervention; one saying it would have ‘*picked them up’* if their spouse could have had a peer mentor (Field Notes C001). One participant allocated to the intervention was relatively dismissive of the intervention saying they ‘*didn’t really need one, so would be better given to someone else*’ (Field Notes P005).

*Matching the peer mentor with a participating patient:* Five of six patients randomised to receive a peer mentor were successfully matched. Given the wide geographical spread of mentors, this was done primarily on the basis of proximity, but with attention to specific requests e.g. on gender or diagnosis of peer mentor. Patient and peer mentor participants were mostly positive about the person they were matched with:

*I felt safe. I did feel really safe and comfortable with [name of mentor]. And, she was pretty good I’d say, she is good, incredibly generous you know, in the way she would listen. Yeah, so it made it very easy I think, for me to just enjoy it. P003 (male, matched with female mentor).*

*Receiving the intervention:* The intervention was delivered in variable ways, as allowed within the protocol. Some peer mentors and patient participants preferred to meet face-to-face:

*But, face-to-face, there are all these little things that are going on and you know, there is the eye contact and there is kind of, you know you pick stuff up that … P003*.

Others primarily interacted remotely by telephone or text:

*Yeah, it was just the once [they met up] wasn’t it, but then we texted and Facebooked, yeah. P005.*

Contact logs were appropriately completed by most mentors. No adverse or serious adverse events were noted. No use of the emergency helpline was triggered.

Quantitative data for our planned primary outcome of quality of life is tabulated below (Table 3). Details of secondary outcomes of health-related quality of life, depression, social support, and carer support are tabulated in Supplementary Tables 1–5. No specific trends in feedback on chosen measures were apparent. By 12 weeks, attrition from the study and/or non-completion of measures was noted.

**Table 3:** Quality of Life Data^a^

	Patients recruited (Control) (*n* = 6)	Patients recruited (Intervention) (*n* = 6)	Carer of P Control recruited (*n* = 4)	Carer of P Intervention recruited (*n* = 2)	Mentor Matched (*n* = 5)
*WHOQOL-BREF Physical*					
T0					
Mean (SD)	60.61 (24.52)	54.16 (21.35)	49.40 (5.95)	78.57 (5.05)	72.85 (16.86)
Median (IQR)	73.21 (43.90)	46.42 (40.18)	46.42 (8.93)	78.57 ()	78.57 (32.14)
Range	20.83–78.57	28.57–82.14	46.43–58.33	75–82.14	50–89.29
Missing	0	0	0	0	0
T4					
Mean (SD)	47.61 (23.74)	59.67 (29.54)			
Median (IQR)	39.28 (43.45)	55.35 (55.80)			
Range	16.67–75	32.14–95.83			
Missing	1	2			
T12					
Mean (SD)	21.42 (7.14)	62.50 (28.04)	78.57 (15.15)	46.43	69.64 (17.12)
Median (IQR)	21.42 ()	58.92 (51.79)	78.57 ()	xx	73.21 (32.14)
Range	14.29–28.57	32.14–100	67.86–89.29	xx	46.43–85.71
Missing	3	2	2	0	1
*WHOQOL-BREF Psychological*					
T0					
Mean (SD)	70.13 (10)	75.69 (13.79)	62.50 (18.94)	75 (11.78)	74.50 (12.12)
Median (IQR)	72.91 (18.75)	77.08 (25)	64.58 (35.42)	75 ()	75 (22.08)
Range	58.33–83.33	58.33–95.83	37.50–83.33	66.67–83.33	60–91.67
Missing	0	0	0	0	0
T4					
Mean (SD)	65.83 (10.78)	71.87 (17.13)			
Median (IQR)	66.66 (18.75)	72.91 (32.29)			
Range	50–79.10	50–91.67			
Missing	1	2			
T12					
Mean (SD)	47.22 (2.40)	82.29 (9.23)	58.33 (29.46)	64.58 (14.73)	77.08 (13.81)
Median (IQR)	45.83 ()	83.33 (17.71)	58.33 ()	64.58 ()	72.91 (25)
Range	45.83–50	70.83–91.67	37.50–79.17	54.17–75	66.67–95.83
Missing	3	2	2	0	1
*WHOQOL-BREF Social relations*					
T0					
Mean (SD)	66.66 (22.36)	81.25 (18.95)	67.70 (15.35)	83.33 (11.78)	71.66 (27.38)
Median (IQR)	70.83 (22.92)	89.58 (31.25)	66.66 (28.12)	83.33 ()	66.66 (54.17)
Range	25–91.67	50–100	50–87.50	75–91.67	41.67–100
Missing	0	0	0	0	0
T4					
Mean (SD)	61.66 (21.73)	70.83 (14.43)			
Median (IQR)	75 (33.33)	66.66 (25)			
Range	25–75	58.33–91.67			
Missing	1	2			
T12					
Mean (SD)	55.55 (29.26)	77.08 (17.17)	75 (11.78)	79.16 (17.67)	66.66 (22.56)
Median (IQR)	58.33 ()	75 (31.25)	75 ()	79.16 ()	58.33 (37.50)
Range	25–83.33	58.33–100	66.67–83.33	66.67–91.67	50–100
Missing			2	0	1
*WHOQOL-BREF Environment*					
T0					
Mean (SD)	82.29 (8.53)	81.77 (14.44)	77.34 (10.63)	81.25 (13.25)	78.12 (11.89)
Median (IQR)	82.81 (14.84)	84.37 (24.22)	79.68 (19.53)	81.25 ()	84.37 (21.88)
Range	71.88–93.75	59.38–100	62.50–87.50	71.88–90.63	62.50–90.63
Missing	0	0	0	0	0
T4					
Mean (SD)	71.16 (10.91)	71.09 (26.56)			
Median (IQR)	71.87 (17.41)	76.56 (49.22)			
Range	53.13–81.25	34.38–96.88			
Missing	1	2			
T12					
Mean (SD)	57.14 (9.79)	76.56 (29.14)	71.87 (13.25)	89.06 (11.04)	79.68 (13.85)
Median (IQR)	59.37 ()	85.93 (51.56)	71.87 ()	89.06 ()	78.12 (26.56)
Range	46.43–65.63	34.38–100	62.50–81.25	81.25–96.88	65.63–96.88
Missing	3	2	2	0	1

^a^A higher score indicates a better quality of life. Empty parentheses () for IQR values indicates fewer than 4 observations, hence insufficient variability to compute an IQR. xx means a constant value, for example two with the same value, so there is no median or range

Although extreme caution is required in any analysis and interpretation of these data due to the small numbers, and potential for outliers, it appears that for the WHOQOL-BREF, those in the intervention group experienced an improvement in quality of life, and those in the control group a decline, in most domains. Proposed secondary outcomes for any future main trial included quality of life measured using the Quality of Life Data (QLQ-C15-PAL), where quality of life declined for all, but more steeply for those in the control group. Similar trends of a potential effect on depression are seen, but with little effect indicated on coping, social or carer support (Supplementary Tables 1–5).

While these are small data sets, it is worth noting that there is substantial variability in scores. This indicates that there were no floor or ceiling effects evident, and provides some indication that chosen measures are appropriate. Whilst specific cancer symptoms appeared to deteriorate over time, as may be expected in a cohort of people with advanced cancer, the WHOQOL-BREF detects potential improvement in general aspects of qualify of life on all domains. Quantitative data resonate with the sentiments from the qualitative data.

Qualitative data collected from mentors and participants indicated satisfaction with the peer mentor concept, and benefits perceived from the interaction, for both peer mentors, patient and carer participants. Some indicated conversations they were unlikely to have with others, whether family, friends, or health care professionals:

*I was quite surprised by the level of the in-depth conversations that we had so they were really good and he probably, yeah, he genuinely would be the only person I have talked to about spending time thinking about dying, he and I have both spent time thinking about dying and, you know, it’s almost like it had to be somebody else who’s been in that situation and I was okay to talk about. And much as it’s lovely to have family and friends who are supportive, you just know that they don’t want to know that you’ve spent time thinking about dying because they’re trying to look after you and keep you alive and everything. M505*

Informal conversations were perceived as helpful, enabling tacit permission to discuss cancer issues if required:

*I think the conversation changed in respect of we got the business of why we were meeting out of the way, so we knew about each other's diagnosis. We knew a little bit about each other's background. So from then on when we met it was more as friends … But it was more a genial, chatty conversation, more like a friendship, but obviously a friendship that’s got specific advantages if need be. So there was always that understanding that, if need be, the topic could change to cancer or to treatment or to something … so yes, we did talk about dying, making the most out of life, and I think it helped we were quite similar in our outlook. So again, that probably contributed to why we got on so well M506*

There could be challenges however, as even when people ‘gelled’ personally, they could have different outlooks on life:

*I was shocked and stunned at the fact that actually we gelled quite quickly … I thought they were great, they’re lovely people. But I really did a couple of times want to go to [name of patient], grab him by the collar and shake him, and ‘go get your boxing gloves on son', you know? … Was basically to water that down, that attitude down, and then I suppose a couple of things that I did do or did say for them they benefitted from. M510.*

## Discussion

This feasibility study of a novel peer mentor intervention identified that recruiting patient participants to such an intervention, primarily through out-patient oncology clinic settings, was not feasible. Indications are, however, that it is possible to recruit and train peer mentors, and that once recruited to the study the intervention and study processes are feasible. The participants who received a peer mentor reported satisfaction from the intervention, and the quantitative data are indicative of possible benefit. Missing data and participant attrition were minimal at 4 weeks, but ill health or death caused some attrition and missing data at 12 weeks. If alternative modes of patient participant recruitment were tested and successful, it is likely that a full trial of this intervention would be possible.

This study reinforces the known value of feasibility studies [37]. Assumptions had been made that patient participant recruitment would not necessarily be the concern in this study, with feasibility objectives focused more on development of the intervention, recruiting peer mentors, and other study processes. Patients with identical characteristics had been successfully recruited to our prior qualitative study using the same criteria, the same settings, and in many cases, the same research nurses [22]. Recruitment processes and materials had been carefully planned with input from our patient and public representatives and research nurses, as is best practice and known to improve recruitment [38]. The intervention had been developed from clear recommendations from both our own research, and from the best available evidence [39]. Amendments were implemented swiftly, taking account of evidence on barriers and facilitators to recruitment to palliative care trials [40].

However, these measures were insufficient to improve patient recruitment. Four factors are hypothesised to have had an effect. First, some clinicians may have been nervous about an intervention provided by peers, and concerned about enabling identification of eligible participants. There may be a sense of professional ‘ownership’ of patients, and anecdotally some clinicians did not enable recruitment from their clinics because they expressed concerns about who the peer mentors were, their training, and the safety of the intervention. Peer mentor interventions with successful recruitment often are those that provide the intervention in the same setting as the recruiting clinicians, and may enable familiarity with those providing peer mentoring [41, 42]. Second, clinics may not be appropriate places to recruit for a non-medical intervention, especially in oncology clinics in large cancer centres where there may be other ongoing trials, where recruitment effort from staff may be focused on other studies. Clinics are busy environments, with a perceived focus on consultations, results and care planning. Many patients took an information pack, but never responded to the research team. Third, it may be difficult to admit that peer support could potentially be helpful in a clinic environment when many attend with those who already provide support. Efforts were made to emphasise that peer support is different, and complementary to, existing forms of support, but this may be a reason why people declined to take an information pack. Fourth, participating in a peer mentor intervention may not meet people’s perceived needs, which may explain why some took a pack but never responded. It is likely that peer mentor interventions may not be welcomed by, nor suitable for, all those with advanced cancer.

Recruiting for a community delivered, peer mentor intervention may be more effective conducted outside clinical, or at least hospital, settings. Social and traditional media advertising proved effective as a mode of recruiting peer mentors themselves, and it may be that such avenues could be explored for those receiving the intervention, as well as those trained to be a mentor. This would be congruent with the mutuality of a peer mentor intervention. Hospices were keen to recruit, but the timescales mitigated against this form of recruitment. General practice or community nursing services may also have contact with patients who may be interested in such interventions.

Whilst we do not have the full data set as planned, there were no indications from participants who did take part or from data collected, that there would be issues with delivering the intervention as planned or collecting planned evaluation data. A tighter geographical location would need to be considered in any future studies, as geography proved the main factor in matching peer mentors and patients due to our wide catchment area. Peer mentor recruitment and training was unproblematic, and patients and mentors reinforced the importance of ‘getting on’ with each other rather than being matched on a narrower range of criteria [25].

Whilst only a small proportion of participants had left the study by week 12, we did have missing data at that point, usually due to deterioration in health status. It may be that collecting data at 4 weeks and 8 weeks may produce a more complete data set. There were no adverse events reported, and participants and mentors reported satisfaction with procedures. Peer mentor interventions are not, however, risk free. Study sponsors and providers must be willing to acknowledge and accept some risk.

Interventions influencing quality of life with this group of patients are important. The low, and often deteriorating, quality of life of the small cohort studied mirrors that seen in other palliative care studies [43, 44]. Yet for those who received the intervention, other more general aspects of quality of life assessed by the WHOQOL-BREF seemed to improve. Volunteering, befriending and peer-mentor type interventions remain potentially important, with what evidence there is pointing to potential effect [20]. Such interventions also have policy relevance, matching the current focus on living with and beyond cancer, and the importance of self-management [45].

## Conclusions

Despite the challenges of this feasibility study, it is likely that such interventions hold promise for further study if the practical and methodological issues demonstrated here can be addressed. Such a study is likely to be a pragmatic trial, where the effectiveness of an intervention is tested in real-world routine practice, given that it is likely inappropriate to further specify the peer-delivered intervention [46].

Recommendations for future studies include:

- Recruiting patient participants from a wide variety of sources, including through social and traditional media.
- Measuring a narrower range of potential outcomes, with a focus on a generic, broad quality of life measure.
- Measuring the primary outcome at around 4–6 weeks after intervention inception.
- Matching mentors on geography and following indications of ‘getting on’, rather than other factors is likely to be appropriate.
- Writing a flexible intervention specification that allows for difference in mode and frequency of contact.
- Enabling those referring to the peer mentor intervention to meet trained peer mentors.
- Using pragmatic trial designs such as wait-list or stepped wedge, to enable all those referred to experience the intervention, once it has commenced [47, 48]. Patient preference designs could also be considered, as people may not be in equipoise about the intervention [49, 50].

## Additional File

**Additional File 1.** Supplementary Data file including tables Quality of Life Data (QLQ-C15-PAL), Depression PHQ 9, Social Support Scale: mMOS-SS, BriefCOPE data, CSNAT data.

## Data Availability

The datasets used and/or analysed during the current study are available from the corresponding author on reasonable request.

## Abbreviations

WHOQOL-BREF: World Health Organisation Quality of Life Assessment short-form
EORTC QLQ C 15 PAL: European Organisation for the Research and Treatment of Cancer Quality of Life Questionnaire Cancer Palliative Care
PHQ-9: Patient Health Questionnaire
mMOS-SS: Modified Medical Outcomes Study Social Support Survey
CSNAT: Carer Support Needs Assessment Tool
CTU: Clinical Trials Unit
