# Supplementary material for: Peer support to maintain psychological wellbeing in people with advanced cancer: findings from a feasibility study for a randomised controlled trial
Source: BMC Palliat Care. 2020 Aug 17;19:129. doi: 10.1186/s12904-020-00631-z (PMC7433175; doi:10.1186/s12904-020-00631-z)
Supplement: Supplementary file 1 — Additional File 1. Supplementary Data file including tables Quality of Life Data (QLQ-C15-PAL), Depression PHQ 9, Social Support Scale: mMOS-SS, BriefCOPE data, CSNAT data. [file 12904_2020_631_MOESM1_ESM.docx]

**Supplementary data**

Supplementary Table 1 Quality of Life Data (QLQ-C15-PAL)

|  | Patients  recruited  (Control)  (n=6) | Patients  recruited (Intervention)  (n=6) |
| --- | --- | --- |
| *QLQ-C15-PAL Physical Functioning* | | |
| T0  Mean (SD)  Median (IQR)  Range  Missing | 55.55 (32.56)  60 (43.33)  0 – 93.33  0 | 58.88 (27.46)  66.66 (48.33)  20 – 93.33  0 |
| T4  Mean (SD)  Median (IQR)  Range  Missing | 54.66 (38.12)  46.66 (73.33)  6.67 – 93.33  1 | 61.66 (24.57)  60 (45)  33.33 – 93.33  2 |
| T12  Mean (SD)  Median (IQR)  Range  Missing | 24.44 (23.41)  26.66 ()  0 – 46.67  3 | 68.33 (16.66)  60 (25)  60 – 93.33  2 |
| *QLQ-C15-PAL Fatigue* | | |
| T0  Mean (SD)  Median (IQR)  Range  Missing | 42.59 (39.38)  44.44 (75)  0 – 100  0 | 59.25 (32.71)  66.66 (33.33)  0 – 100  0 |
| T4  Mean (SD)  Median (IQR)  Range  Missing | 51.11 (34.78)  66.66 (61.11)  0 – 88.89  1 | 55.55 (38.49)  66.66 (66.67)  0 – 88.89  2 |
| T12  Mean (SD)  Median (IQR)  Range  Missing | 88.88 (19.24)  100 ()  66.67 – 100  3 | 50 (43.03)  50 (83.33)  0 – 100  2 |
| *QLQ-C15-PAL Nausea* | | |
| T0  Mean (SD)  Median (IQR)  Range  Missing | 8.33 (20.41)  0 (12.50)  0 – 50  0 | 5.55 (8.60)  0 (12.50)  0 – 16.67  0 |
| T4  Mean (SD)  Median (IQR)  Range  Missing | 13.33 (21.73)  0 (33.33)  0 – 50  1 | 16.66 (23.57)  8.33 (41.67)  0 – 50  2 |
| T12  Mean (SD)  Median (IQR)  Range  Missing | 38.88 (19.24)  50 ()  16.67 – 50 | 8.33 (9.62)  8.33 (16.67)  0 – 16.67 |
| *QLQ-C15-PAL Emotional Functioning* | | |
| T0  Mean (SD)  Median (IQR)  Range  Missing | 68.05 (33.50)  75 (64.58)  16.67 – 100  0 | 80.55 (12.54)  83.33 (20.83)  66.67 – 100  0 |
| T4  Mean (SD)  Median (IQR)  Range  Missing | 65 (22.36)  66.66 (37.50)  41.67 – 100  1 | 70.83 (15.95)  75 (29.17)  50 – 83.33  2 |
| T12  Mean (SD)  Median (IQR)  Range  Missing | 22.22 (25.45)  16.66 ()  0 – 50  3 | 79.16 (25)  83.33 (45.83)  50 – 100  2 |
| *QLQ-C15-PAL Quality of Life* | | |
| T0  Mean (SD)  Median (IQR)  Range  Missing | 63.88 (19.48)  66.66 (37.50)  33.33 – 83.33  0 | 72.22 (27.21)  75 (54.17)  33.33 – 100  0 |
| T4  Mean (SD)  Median (IQR)  Range  Missing | 66.66 (11.78)  66.66 (16.67)  50 – 83.33  1 | 66.66 (30.42)  66.66 (58.33)  33.33 – 100  2 |
| T12  Mean (SD)  Median (IQR)  Range  Missing | 27.77 (9.62)  33.33 ()  16.67 – 33.33  3 | 58.33 (34.69)  58.33 (66.67)  16.67 – 100  2 |

Empty parentheses () for IQR values indicates fewer than 4 observations, hence insufficient variability to compute an IQR. xx means a constant value, for example two with the same value, so there is no median or range.

Supplementary Table 2 Depression score: PHQ-9

|  | Patients  recruited  (Control)  (n=6) | Patients  recruited (Intervention)  (n=6) |
| --- | --- | --- |
| *PHQ-9* | | |
| T0  Mean (SD)  Median (IQR)  Range  Missing | 6.50 (4.76)  7 (9.75)  0 – 12  0 | 4 (3.84)  3 (5)  0 – 11  0 |
| T4  Mean (SD)  Median (IQR)  Range  Missing | 5.60 (3.36)  7 (5.50)  0 – 8  1 | 5.75 (5.31)  5.50 (10.25)  0 – 12  2 |
| T12  Mean (SD)  Median (IQR)  Range  Missing | 15 (1.73)  16 ()  13 – 16  3 | 4.5 (4.12)  5 (7.50)  0 – 8  2 |

Higher scores indicate more severe depression, with scores of 0-4 indicating none, 5-9 mild, 10-14 moderate, 15-19 moderately severe, and 20-27 severe depression. Empty parentheses () for IQR values indicates fewer than 4 observations, hence insufficient variability to compute an IQR. xx means a constant value, for example two with the same value, so there is no median or range.

Supplementary Table 3 Coping score: BriefCOPE

|  | Patients  recruited  (Control)  (n=6) | Patients  recruited (Intervention)  (n=6) | Carer of P Control  recruited  (n=4) | Carer of P Intervention  recruited  (n=2) | Mentor  recruited  (n=5) |
| --- | --- | --- | --- | --- | --- |
| *BriefCOPE Problem-focused coping* | | | | | |
| T0  Mean (SD)  Median (IQR)  Range  Missing | 15 (5.29)  15 (10.50)  9 – 21  0 | 13.83 (5.70)  12.50 (8)  6 – 23  0 | 16.75 (4.57)  18.50 (7.75)  10 – 20  0 | 13 (4.24)  13 ()  10 – 16  0 | 18.20 (1.30)  18 (2.50)  17 – 20  0 |
| T4  Mean (SD)  Median (IQR)  Range  Missing | 13.60 (7.02)  11 (13.50)  6 – 22  1 | 12.75 (6.07)  12.50 (11.75)  6 – 20  2 |  |  |  |
| T12  Mean (SD)  Median (IQR)  Range  Missing | 15 (3.60)  16 ()  11 – 18  3 | 12 (7.65)  10 (14)  6 – 22  2 | 17 (1.41)  17 ()  16 – 18  2 | 11.50 (0.70)  11.50 ()  11 - 12  0 | 11.25 (5.56)  11 (10.25)  6 – 17  1 |
| *BriefCOPE Emotion-focused coping* | | | | | |
| T0  Mean (SD)  Median (IQR)  Range  Missing | 25.83 (6.55)  27.50 (12.50)  17 – 34  0 | 22 (6.72)  21.50 (8.75)  14 – 34  0 | 25 (3.91)  25.50 (7.50)  20 – 29  0 | 18.50 (3.53)  18.50 ()  16 – 21  0 | 26 (7.07)  24 (13)  17 – 35  0 |
| T4  Mean (SD)  Median (IQR)  Range  Missing | 22.20 (8.34)  27 (15)  10 – 29  1 | 20.25 (6.13)  18 (10.75)  16 – 29  2 |  |  |  |
| T12  Mean (SD)  Median (IQR)  Range  Missing | 28.66 (1.52)  29 ()  27 – 30  3 | 19.50 (8.66)  18.50 (16.50)  11 – 30  2 | 24 (4.24)  24 ()  21 – 27  2 | 19 (xx)  19 ()  19  0 | 18 (9.41)  16 (17.50)  10 – 30  1 |
| *BriefCOPE Dysfunctional coping* | | | | | |
| T0  Mean (SD)  Median (IQR)  Range  Missing | 19.16 (3.31)  18.50 (4.50)  16 – 25  0 | 17.50 (2.34)  18 (1.75)  13 – 20  0 | 25 (8.36)  23 (15.50)  18 – 36  0 | 14 (xx)  14 ()  14  0 | 18.40 (5.12)  17 (9.50)  12 – 25  0 |
| T4  Mean (SD)  Median (IQR)  Range  Missing | 18.20 (6.53)  16 (10.50)  12 – 29  1 | 19 (2.94)  19 (5.50)  16 – 22  2 |  |  |  |
| T12  Mean (SD)  Median (IQR)  Range  Missing | 19.33 (5.85)  17 ()  15 – 26 | 15.75 (2.21)  16 (4.25)  13 - 18 | 23.50 (16.26)  23.50 ()  12 – 35  2 | 14.50 (2.12)  14.50 ()  13 – 16  0 | 16.75 (5.18)  16 (9.75)  12 – 23  1 |

Empty parentheses () for IQR values indicates fewer than 4 observations, hence insufficient variability to compute an IQR. xx means a constant value, for example two with the same value, so there is no median or range.

Supplementary Table 4 Social Support Scale: mMOS-SS

|  | Patients  recruited  (Control)  (n=6) | Patients  recruited (Intervention)  (n=6) |
| --- | --- | --- |
| *mMOS-SS Instrumental* | | |
| T0  Mean (SD)  Median (IQR)  Range  Missing | 3.29 (1.29)  3.50 (2.19)  1.25 – 4.75  0 | 2.58 (1.64)  2.25 (3.25)  1 – 5  0 |
| T4  Mean (SD)  Median (IQR)  Range  Missing | 3.65 (1.16)  4 (2.13)  2 – 5  1 | 3.75 (1.89)  4.50 (3.25)  1 – 5  2 |
| T12  Mean (SD)  Median (IQR)  Range  Missing | 4.66 (0.38)  4.75 ()  4.25 – 5  3 | 3.25 (1.70)  3.50 (3.25)  1 – 5  2 |
| *mMOS-SS Emotional* | | |
| T0  Mean (SD)  Median (IQR)  Range  Missing | 4.08 (0.81)  4 (1.63)  3 – 5  0 | 3.95 (1.43)  4.62 (2.38)  1.50 – 5  0 |
| T4  Mean (SD)  Median (IQR)  Range  Missing | 4.15 (0.94)  4.25 (1.75)  2.75 – 5  1 | 4.50 (0.45)  4.50 (0.88)  4 – 5  2 |
| T12  Mean (SD)  Median (IQR)  Range  Missing | 4.25 (1.08)  4.75 ()  3 – 5  3 | 4.31 (0.94)  4.62 (1.69)  3 – 5  2 |
| *mMOS-SS Total* | | |
| T0  Mean (SD)  Median (IQR)  Range  Missing | 3.68 (0.87)  3.75 (1.78)  2.75 – 4.63  0 | 3.27 (1.33)  3.25 (2.72)  1.50 – 5  0 |
| T4  Mean (SD)  Median (IQR)  Range  Missing | 3.90 (1.04)  4.12 (1.94)  2.38 – 5  1 | 4.12 (1.09)  4.43 (2)  2.63 – 5  2 |
| T12  Mean (SD)  Median (IQR)  Range  Missing | 4.45 (0.73)  4.75 ()  3.63 – 5  3 | 3.78 (0.97)  3.56 (1.78)  3 – 5  2 |

Empty parentheses () for IQR values indicates fewer than 4 observations, hence insufficient variability to compute an IQR. xx means a constant value, for example two with the same value, so there is no median or range.

Supplementary Table 5 Carer support: CSNAT

|  | Carer of P Control  recruited  (n=4) | Carer of P Intervention  recruited  (n=2) |
| --- | --- | --- |
| *CSNAT* | | |
| T0  Mean (SD)  Median (IQR)  Range  Missing | 14.50 (6.55)  12.50 (11.50)  9 – 24  0 | 1.50 (2.12)  1.50 (5)  0 – 3  0 |
| T12  Mean (SD)  Median (IQR)  Range  Missing | 12 (9.89)  12 ()  5 – 19  2 | 1 (1.41)  1 ()  0 – 2  0 |

Empty parentheses () for IQR values indicates fewer than 4 observations, hence insufficient variability to compute an IQR. xx means a constant value, for example two with the same value, so there is no median or range.
